# Whole-Genome Re-Sequencing Reveals Genetic Diversity and Population History of Arunachali Mithun (Bos frontalis)

**DOI:** 10.3390/ijms27135824

**Published:** 2026-06-27

**Authors:** Kuluve Chotso, Hanumant S. Rathore, Harshit Kumar, Jayanta Kumar Chamuah, Sapunii S. Hanah, Girish Patil Shivanagowda

**Affiliations:** 1 Department of Biotechnology, Nagaland University, Kohima 797004, Nagaland, India; 2 ICAR-National Research Centre on Mithun, Medziphema, Chümoukedima 797106, Nagaland, India

**Keywords:** Mithun, *Bos frontalis*, genetic diversity, runs of homozygosity, inbreeding, linkage disequilibrium, effective population size, nucleotide diversity, heterozygosity

## Abstract

The Arunachali mithun (*Bos frontalis*) is a semi-domesticated bovine of profound cultural and economic significance to the indigenous Arunachali tribal communities of Northeastern India, yet it remains among the least genomically characterised large ruminants, leaving its conservation status without an empirical genetic foundation. We performed whole-genome re-sequencing (~10× coverage) of 11 individuals and analysed 4,943,593 high-quality biallelic single nucleotide polymorphisms (SNPs) after stringent quality control. Genome-wide mean observed heterozygosity (H_o_ = 0.2854), expected heterozygosity (H_e_ = 0.3347), and nucleotide diversity (π = 7.16 × 10^−4^) revealed moderate genetic diversity, substantially lower than that of related commercial bovine species. A consistent heterozygosity deficit (H_o_ − H_e_ = −0.0493) and the convergence of four independent inbreeding coefficients around 0.143–0.147 indicated moderate inbreeding of predominantly reflecting an ancient origin, corroborated by runs of homozygosity (ROH) analysis in which 93.2% of 24,937 detected segments fell in the short length class (100–250 kb). Linkage disequilibrium decayed from r^2^ ≈ 0.57 at <100 kb to a plateau of r^2^ ≈ 0.33 beyond 4–5 Mb, consistent with a small effective population size (N_e_) declining from approximately 101,850 (~2228 generations ago) to approximately 160 (~5 generations ago), with ab Ne of approximately 3865 at ~100 generations ago and 423 at ~10 generations ago. These findings establish a whole-genome-based genetic diversity baseline for the Arunachali mithun and provide actionable genomic evidence for conservation and managed breeding interventions.

## 1. Introduction

The mithun (*Bos frontalis*) or gayal is a large semi-domesticated bovine native to the tropical and subtropical forests of Northeastern India, Bhutan, Myanmar, and southern China [1,2,3]. The Arunachali mithun presents deep cultural, social, and economic value to the indigenous tribal communities of Arunachal Pradesh. It is simultaneously a medium of marital exchange, a symbol of social status, and an important source of animal protein, primarily through its meat, and to a lesser extent, milk to tribal communities. Compared with other large ruminants, the mithun is one of the least genetically characterised species with most research conducted on characterising the phenotype, mitochondrial phylogenetic trees, and small-scale microsatellite studies. At present, the conservation of rare and indigenous livestock breeds is a global priority, explicitly mandated under the United Nations Sustainable Development Goal 2 (SDG 2: Zero Hunger), Target 2.5, which calls for the maintenance of the genetic diversity of farmed and domesticated animals, monitored through SDG indicators 2.5.1 (genetic resources secured in conservation facilities) and 2.5.2 (proportion of local breeds classified as being at risk of extinction) [4]. This is because there is a loss of genetic diversity due to inbreeding, destruction of habitats, and the contracting population size of the mithun [5,6,7]. 

Among the principal threats to the genetic viability of small, isolated populations, inbreeding and the associated reduction in the effective population size (N_e_) are of particular concern. When populations become small and reproductively isolated, mating between related individuals becomes unavoidable, increasing the proportion of the genome that is autozygous (identical by descent at both alleles), thereby elevating inbreeding coefficients across generations [8,9]. This process manifests genomically as an excess of runs of homozygosity (ROH), defined as long contiguous segments of identical homozygous genotypes, whose length distribution encodes the timing and intensity of past inbreeding events [10,11]. The Arunachali mithun is particularly vulnerable to these dynamics: its semi-domesticated management system, in which animals roam freely within village territories with limited inter-herd exchange, structurally promotes non-random mating within geographically fragmented herds [3]. Combined with the complete absence of pedigree records and the disproportionate use of a limited number of breeding bulls (a pattern documented in other managed indigenous bovine populations), these husbandry practices create conditions under which inbreeding can accumulate undetected across generations, silently eroding the genetic diversity of a species that is already characterised by a small historical N_e_ [3,6]. Despite this conservation urgency, no genome-wide assessment of inbreeding levels, population structure, or historical demographic trajectories has hitherto been conducted for the Arunachali population, leaving conservation managers without the empirical genomic baseline necessary to design evidence-based interventions.

Effective genetic management of such breeds requires detailed knowledge of population structure, levels of genetic diversity, the extent of inbreeding, and historical demographic trajectories [12]. Whole-genome sequencing (WGS) now provides the resolution necessary to characterise these parameters with unprecedented precision, enabling the detection of millions of single nucleotide polymorphisms (SNPs) distributed evenly across the entire genome [13]. Crucially, unlike previous foundational studies on the mithun that relied on commercial high-density SNP arrays, WGS overcomes the SNP ascertainment bias inherent to array platforms. This allows for the capture of rare, species-specific variants and provides the unascertained, base-pair resolution required for fine-scale demographic modelling and true nucleotide diversity estimation.

The present study directly addresses this gap by deploying whole-genome resequencing (WGS) to quantify, at single-nucleotide resolution, the breadth and depth of genetic diversity, the extent and origin of inbreeding, and the trajectory of the effective population size decline in Arunachali mithun. Using a dataset of 11 individuals sequenced against the *Bos frontalis* YUN_Gayal_1 reference genome (GCA_043643345.1), we quantify heterozygosity, nucleotide diversity, Hardy–Weinberg equilibrium, minor allele frequency distribution, pairwise genetic distances, inbreeding coefficients (F_ROH_, F_HOM_, F_UNI_, F_GRM_), LD decay, haplotype block structure, and historical effective population size. Together, these analyses provide the first WGS-derived genomic framework for the conservation and sustainable management of this culturally irreplaceable indigenous bovine, delivering a critical genomic landscape for conservation planning and future selective breeding programmes.

## 2. Results

### 2.1. Variant Calling and Quality Control

Following GATK HaplotypeCaller-based joint genotyping [14] across all 11 Arunachali mithun individuals, a total of approximately 12.61 million raw SNPs were identified. After applying GATK hard filters and PLINK population-level quality control (MAF ≥ 0.05, HWE *p* ≥ 1 × 10^−5^, genotype missingness < 5%) [15], a total of 4,943,593 high-quality biallelic SNPs distributed across all autosomes were retained. The HWE filter removed no variants (0 SNPs), indicating that allele frequency distributions across the dataset are largely consistent with Hardy–Weinberg expectations, with the exception of a minor subset of SNPs at very low *p*-values (Figure S1).

### 2.2. ADMIXTURE Analysis of the Arunachali Mithun

**Figure 1:**
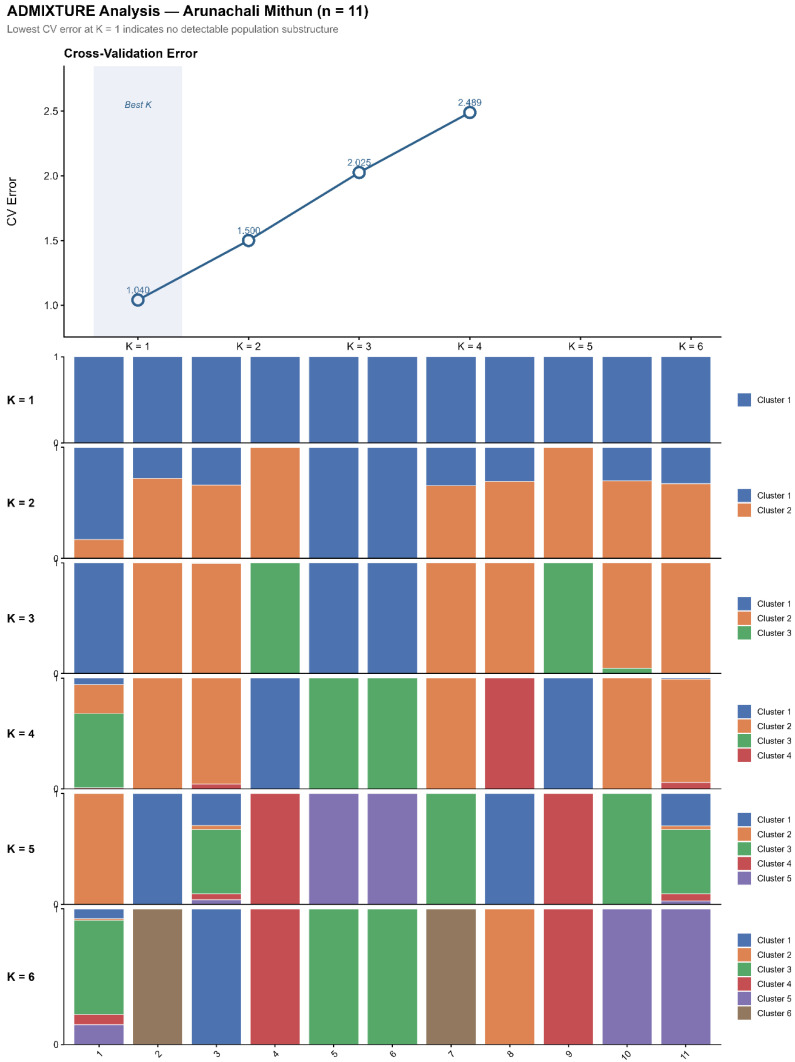
ADMIXTURE analysis of Arunachali mithun (n  =  11) based on 117,409 LD-pruned SNPs. Upper panel: cross-validation (CV) error for K  =  1 to 6; the lowest CV error at K  =  1 (CV  =  1.040) indicates no detectable population substructure; K  =  5 and K  =  6 failed to converge. Lower panels: ancestry proportion bar plots for K  =  1 to 6; each bar represents one individual and each colour one ancestral cluster. The monotonically increasing CV error and absence of stable cluster assignments at K  >  1 confirm K  =  1 as the best-supported model.

To investigate the genetic structure of the Arunachali mithun, we performed an ADMIXTURE analysis on the 11 sampled individuals utilising 117,409 LD-pruned SNPs. We evaluated potential ancestral clusters ranging from K = 1 to K = 6. The lowest cross-validation (CV) error was observed at K = 1 (CV = 1.040), indicating no detectable population substructure within the cohort. Models assuming higher numbers of ancestral populations (K > 1) yielded monotonically increasing CV errors and lacked stable cluster assignments, with the K = 5 and K = 6 models failing to converge. Consequently, K = 1 is the best-supported model, consistent with the Arunachali mithun sample forming a single panmictic cluster under current sampling; note that statistical power to detect subtle substructure is limited at *n* = 11 (see Section 3.8, Figure 1).

### 2.3. Minor Allele Frequency Distribution

Minor allele frequencies from the 4.94 million QC-passed SNPs presented a typical L-shaped distribution (Figure S2), with the majority of SNPs found in the lowest MAF interval (0.05–0.10), which contained approximately 25 SNPs per unit density. The distribution gradually decreased through the intermediate intervals but still showed several distinct peaks at MAF levels of approximately 0.09, 0.14, 0.18, 0.23, 0.27, 0.32, 0.36, 0.41, and 0.45. These discrete ‘spikes’ in the MAF density distribution correspond to the multi-modal pattern.

### 2.4. Hardy–Weinberg Equilibrium and Heterozygosity

The distribution of HWE *p*-values showed that the vast majority of SNPs (approximately 2.5 million) had *p*-values at or near 1.0, indicating strong conformity with HWE expectations across the genome (Figure S1). A minority of SNPs had very low *p*-values (<0.001), which may reflect genuine biological deviations due to selection, population structure, or genotyping artefacts, though these constituted a very small proportion of the total SNP set. The scatter plot of observed (H_o_) versus expected (H_e_) heterozygosity showed a positive linear relationship (Figure S3), though with considerable scatter and a consistent pattern of H_o_ falling below the 1:1 line for intermediate to high H_e_ values, indicating an overall heterozygosity deficit across the genome.

Mean H_o_ was 0.2854 ± 0.1620 (SE = 7.28 × 10^−5^) and mean H_e_ was 0.3347 ± 0.1207 (SE = 5.43 × 10^−5^). The difference H_o_ − H_e_ was −0.0493 ± 0.1308, indicating that on average, Mithun individuals exhibit approximately 4.93% fewer heterozygous loci than expected under random mating. This deficit is consistent with moderate levels of inbreeding and/or population substructure [8]. 

### 2.5. Nucleotide Diversity

Nucleotide diversity (π) was estimated across 50 kb sliding windows spanning the entire genome using VCFtools 0.1.17 [16]. After excluding windows with <20 variant sites, a total of 48,438 windows were retained. The genome-wide average was 7.157 × 10^−4^ ± 4.860 × 10^−4^ (SE = 2.21 × 10^−6^) with minimum and maximum values of ~0 and ~0.006 respectively across the windows (see Figure S4). The landscape of genome-wide π displayed a fairly even distribution over all autosomes at window scale, but there were isolated peaks in π reaching levels of almost 0.005–0.006 on chromosomes 1, 2, 15, 21, and 23 (Figure S4). These areas with high π nay be indicative of augmented functional variation or lowered constraint on nucleotide diversity. Per-chromosome mean π values, standard deviations, standard errors, and window counts are provided in Supplementary Table S1.

### 2.6. Pairwise Genetic Distance and Allele Sharing

**Figure 2:**
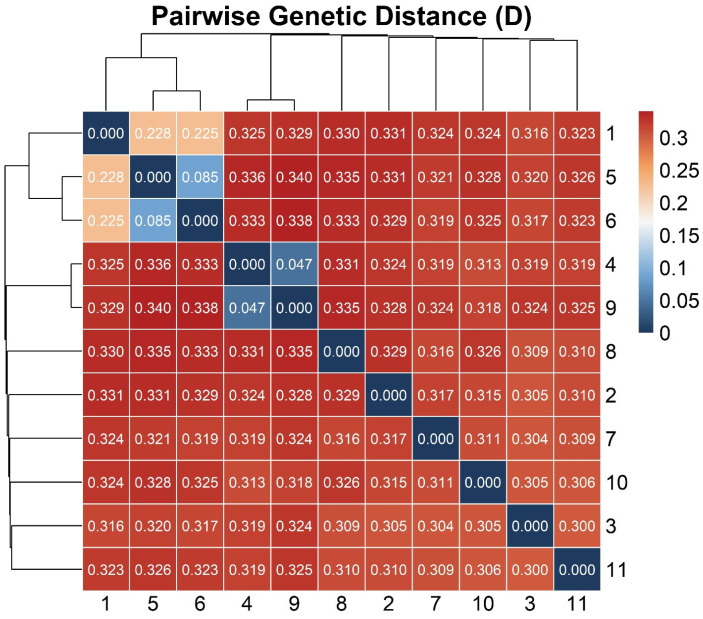
Hierarchically clustered heatmap of pairwise genetic distances (D) among 11 Arunachali mithun individuals. Lower values (blue) indicate higher genomic similarity.

The pairwise genetic distance (D) matrix revealed heterogeneity in genomic relatedness among the 11 Arunachali mithun individuals (Figure 2), with most inter-individual distances falling within a narrow range (D ≈ 0.300–0.340) but two clear outliers standing out from the background. Hierarchical clustering resulted in two major groups: one consisting of individuals 11 and 6, who had a very low pairwise distance (D = 0.047) and hence extremely high genome similarity indicating recent common ancestry or close kinship; and the other nine individuals who constituted a large cluster with inter-individual distances varying from approximately D = 0.300 to D = 0.340. A secondary sub-cluster was conspicuous within this larger cluster for three individuals, who had lower pairwise distances among themselves (D = 0.085–0.228) relative to the rest of the set.

**Figure 3:**
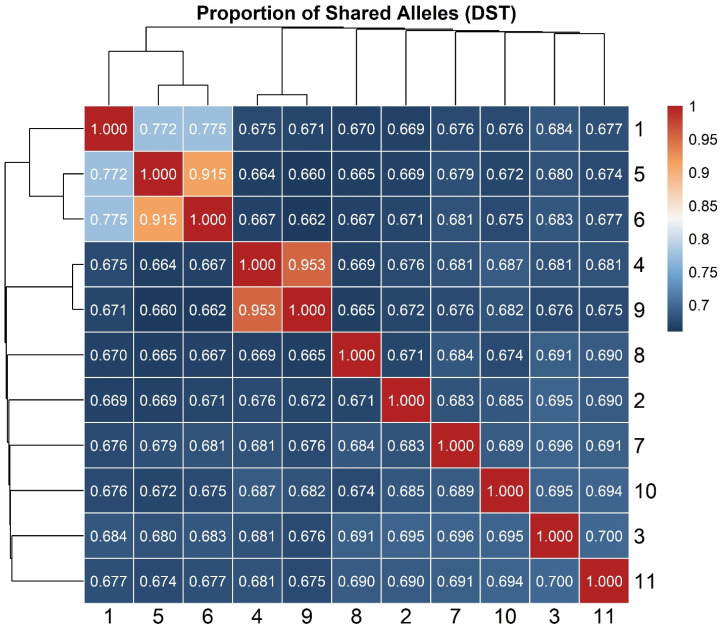
Allele sharing heatmap (D_st_ = 1 − D) showing the proportion of shared alleles between all individual pairs. Higher values (blue) represent greater allele sharing.

The mean pairwise genetic distance across all individual pairs was D = 0.3089 ± 0.0519 (SE = 0.0070), corresponding to a mean allele sharing coefficient of D_ST_ = 0.6911. The complementary allele sharing heatmap (Figure 3) confirmed these patterns, with 11 and 6 sharing 95.3% of alleles and the 1–2–3 sub-cluster sharing 77–91.5% of alleles, substantially higher than the background inter-individual sharing of ~67–70%.

### 2.7. Principal Component Analysis

**Figure 4:**
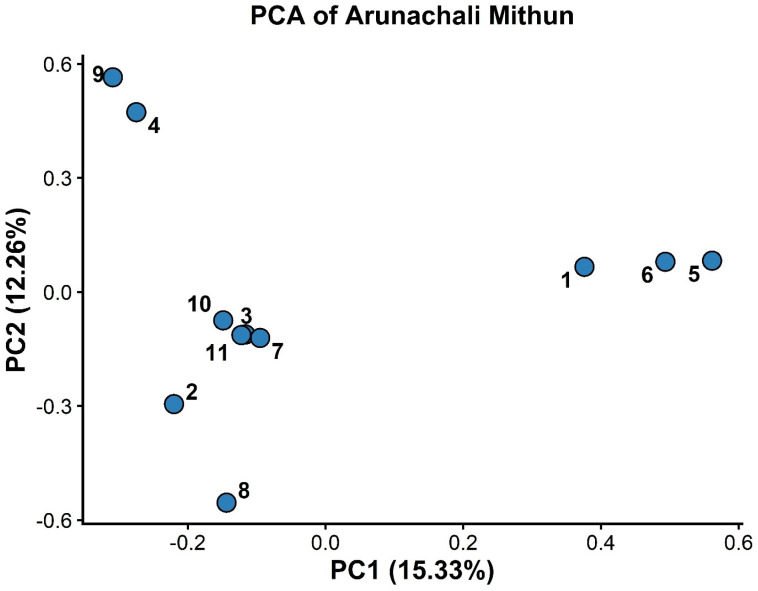
Principal component analysis (PCA) of 11 Arunachali mithun individuals based on ~4.94 million LD-pruned SNPs. PC1 explains 15.33% and PC2 explains 12.26% of total genomic variance. Individual labels correspond to sample IDs; two broad groupings (1–3 vs. the remaining individuals) and the elevated PC2 position of 6/11 are discussed in the text.

The PCA of the 11 Arunachali mithun individuals revealed that the first two principal components together explained 27.59% of the total genomic variance (PC1 = 15.33%; PC2 = 12.26%) (Figure 4). The scatter plot revealed two broad groupings that closely mirrored the relatedness clusters found using the pairwise genetic distance analysis (Section 2.6). The first set was made up of 1, 2, and 3, located on the positive side of PC1 (PC1 ≈ 0.38–0.57), with 2 and 3 very near each other (PC1 ≈ 0.50, PC2 ≈ 0.03) and 1 slightly offset (PC1 ≈ 0.38, PC2 ≈ 0.03). The second and larger set consisting of 4, 5, 6, 7, 8, 9, 10, and 11 was positioned on the negative side of PC1 (PC1 ≈ −0.10 to −0.23), implying a systematic genome-wide differentiation from the 1–3 sub-group. Within this larger cluster, 6 and 11 were found at a significantly higher position on PC2 (PC2 ≈ 0.45–0.58), which is in agreement with their very low pairwise genetic distance (D = 0.047) and high allele sharing (DST = 0.953). Individual 5 was an outlier within the second group on the negative end of PC2 (PC2 ≈ −0.52), suggesting a degree of individual distinctiveness not captured by pairwise distance alone. The spatial positioning of 9 and 11 as the most negative on PC1 within the second group is further consistent with the heatmap evidence that these individuals are among the most genomically differentiated from 1 to 3.

Notably, the PCA pattern provides independent, model-free confirmation of the relatedness signal captured by the pairwise genetic distance and allele sharing. Individuals 11 and 6, with the lowest observed pairwise distance (D = 0.047) and the highest allele sharing (D_st_ = 0.953), are not only very close together on PC2 but also separated from the other individuals, which is a typical signature of closely related individuals inflating the variance captured by a PC axis. Likewise, 2 and 3 (pairwise D = 0.085; D_st_ = 0.915) are almost overlapping in the PCA biplot, which verifies that their genomic similarity is widespread throughout the entire genome rather than being limited to a few isolated segments. Individual 1, while forming a loose sub-cluster with 2 and 3 on PC1, is separated from them on PC2, consistent with its lower but still elevated allele sharing (D_st_ = 0.775 with 3; 0.772 with 2). The convergence of the PCA, pairwise distance, and allele sharing metrics for all flagged pairs substantially strengthens confidence that the detected relatedness is genuine rather than an artefact of a small sample size or LD structure.

### 2.8. Genomic Inbreeding Coefficients

Four complementary genomic inbreeding coefficients were computed to provide a multi-faceted assessment of autozygosity. Remarkably, all four inbreeding estimates converged within a tight range of 0.143–0.147, implying a consistent moderate level of inbreeding signals across all methods. FROH, which derives the measure from the total ROH length relative to the autosomal genome, averaged 0.1445 ± 0.0364 (SE = ±0.011). FHOM, which is based on the homozygosity excess and computed through PLINK–het, averaged 0.1473 ± 0.0783 (SE = ±0.024) [15]. FUNI (GCTA Fhat3), which quantifies the correlation between uniting gametes, averaged 0.1432 ± 0.0892 (SE = ±0.027). FGRM, estimated from the GRM, yielded 0.1432 ± 0.1384 (SE = ±0.042 [17].

**Figure 5:**
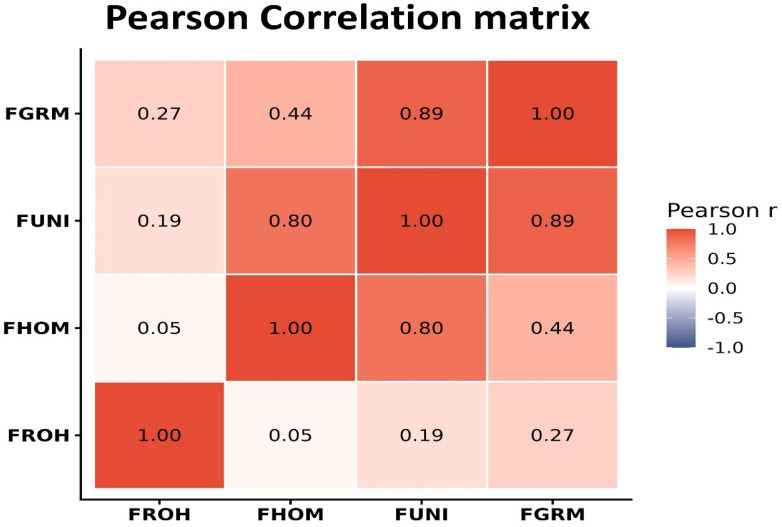
Pearson correlation matrix among four genomic inbreeding coefficients (F_ROH_, F_HOM_, F_UNI_, F_GRM_) in Arunachali mithun. Values represent Pearson r.

The Pearson correlation analysis among the four coefficients (Figure 5) revealed that FUNI and FGRM were most strongly correlated (r = 0.89), followed by FHOM and FUNI (r = 0.80), reflecting the shared methodological basis of the two metrics in allele frequency deviations. By contrast, FROH showed low correlations with the other three estimators (r = 0.05–0.27), which is expected given that FROH captures only segments above the 100 kb minimum length threshold and thus reflects recent-to-moderate inbreeding rather than the genome-wide frequency-based signal captured by the other methods [11,18]. Per-individual values for all 11 individuals are provided in Supplementary Table S2. Pairwise scatter plots among all four coefficients at the individual level are provided in Supplementary Figure S7.

### 2.9. Runs of Homozygosity

ROH analysis identified a total of 24,937 homozygous segments with lengths ≥ 100 kb across all 11 individuals. The length class distribution (Table 1, was strongly dominated by the shortest class (100–250 kb), which contained 23,186 segments (93.2% of all ROH). Importantly, all detected ROH fell within the short ROH category (<1 Mb) by bovine genomic standards, indicating that inbreeding in this population is consistent with a predominance of ancient inbreeding; however, recent inbreeding below the 100 kb detection threshold cannot be excluded given the methodological constraints and the small sample size. The 250–400 kb class contributed 1551 segments (6.2%), and the 400–550 kb class 175 segments (0.7%), all classified as being of ancient origin reflecting long-term drift and founder effects. Only a negligible proportion of ROH fell into the more distant origin classes: 19 segments (0.08%) in the 550–700 kb class and 6 segments (0.02%) in the 700–1000 kb class. The predominance of short ROH is a characteristic genomic signature of inbreeding that accumulated over many generations, as recombination progressively breaks down longer autozygous segments inherited from remote common ancestors into shorter fragments over time [9,10]. The complete absence of ROH ≥ 1 Mb which in cattle genomic studies would indicate moderate to slightly recent inbreeding, suggests that intensive pedigree-based mating has not yet occurred at a detectable genomic scale in the Arunachali mithun.

**Figure 6:**
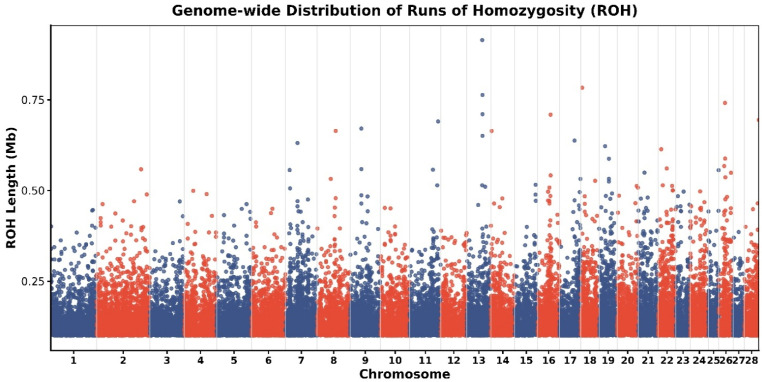
Genome-wide Manhattan plot of ROH segments across all autosomes. Each point represents a single ROH segment coloured alternately by chromosome.

The genome-wide Manhattan plot of ROH (Figure 6) revealed a generally even distribution of ROH segments along all autosomes, with segment lengths concentrated predominantly in the 0.10–0.35 Mb range and rare outlier segments reaching up to 0.9 Mb, particularly on chromosomes 13 and 17. This dispersed pattern indicates that no single chromosomal region is markedly autozygous, and that the inbreeding signal is distributed across the whole genome rather than being localised at specific loci [11,19]. The mean F_ROH_ of 0.1445 is consistent with this observation: while individual ROH segments are small, their cumulative genomic coverage accounts for a substantial proportion of the autosomal genome length.

### 2.10. Linkage Disequilibrium Decay

**Figure 7:**
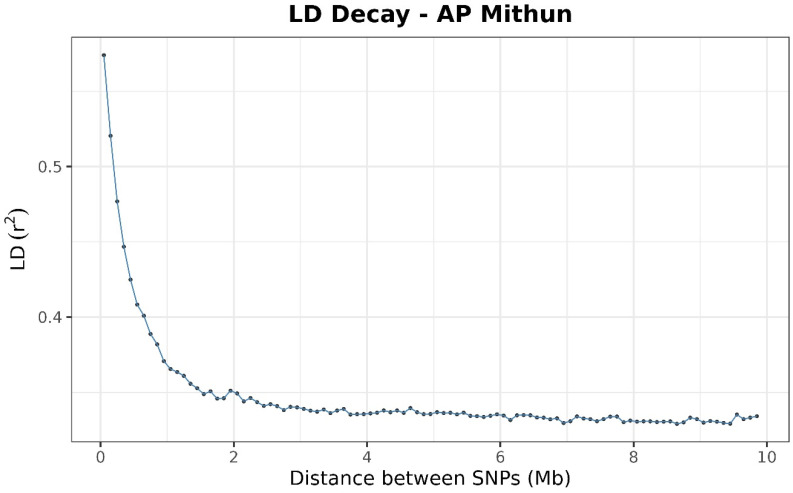
Genome-wide LD decay curve for r^2^ as a function of inter-SNP physical distance (Mb) in Arunachali mithun.

Genome-wide LD was assessed using the r^2^ metric across a range of inter-SNP physical distances [15,20]. The r^2^ decay curve (Figure 7) followed a characteristic exponential decline from an initial value of approximately r^2^ = 0.57 at the shortest distances (<100 kb), declining rapidly to r^2^ ≈ 0.39 by 500 kb and plateauing around r^2^ ≈ 0.33 at distances beyond 4–5 Mb. This finding is characteristic of a small-to-moderate-sized population where LD extends over larger genomic intervals than in large outbred populations [21], a scenario that is very likely the case here.

### 2.11. Effective Population Size

**Figure 8:**
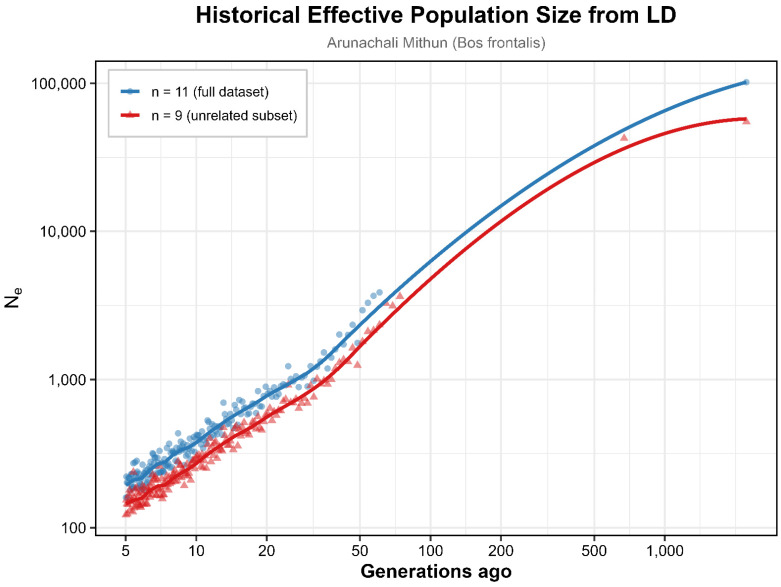
Effective population size (N_e_) estimates as a function of inter-SNP distance bins (corresponding to historical time depth) in Arunachali mithun.

Historical N_e_ was estimated from LD decay data binned by inter-SNP distance, with each distance bin corresponding to a different number of generations in the past t ≈ 1/(2c) [20,21]. The N_e_ estimates declined from approximately 101,850 at the shortest valid distance bin (0–50 kb, reflecting ~2228 generations ago) to approximately 160 at the longest distance bin (~5 generations ago; Figure 8). At key intermediate time depths, N_e_ was approximately 3865 at ~100 generations ago, 1761 at ~50 generations ago, 895 at ~20 generations ago, and 423 at ~10 generations ago, tracing a progressive and sustained contraction in the effective population size. The n = 9 sensitivity analysis (excluding related individuals 3 and 11) yielded consistently lower but directionally identical estimates: N_e_ ≈ 3629 at ~100 generations ago, 1247 at ~50 generations ago, 577 at ~20 generations ago, and 304 at ~10 generations ago, confirming that the historical decline signal is robust to the presence of related individuals. N_e_ estimation was not possible for intermediate time depths (~100–400 generations ago) in the n = 9 dataset, as background LD at those distances approached the sample-size correction threshold (1/n = 0.111), precluding valid r^2^ corrected estimates. The [0, 50] kb bin estimate (N_e_ ≈ 101,850 at ~2228 generations ago) should be interpreted with caution as it is partially affected by the 50 kb pruning window; N_e_ estimates at 5–100 generations ago are the most robust outputs of this analysis. The pattern we observe here traces back to a period of shrinking of the breeding stock, which aligns with the ongoing reduction in the population as a result of domestication bottlenecks and isolation [6,12]. Although the numbers obtained here are lower than the typical Ne trajectories reported for commercial taurine breeds [22], they are broadly in agreement with those of other native Asian cattle populations [3]. These estimates should be interpreted as exploratory order-of-magnitude indicators. Small sample sizes (n = 11) and the presence of related individuals are known to inflate LD-based N_e_ estimates [21], so the absolute values carry substantial uncertainty even though the directional trend of contraction is robust.

### 2.12. Sensitivity Analysis of Unrelated Individuals

To ensure that the identified related individuals (3 and 11) did not disproportionately bias population-level metrics, a sensitivity analysis was performed by repeating all primary analyses on an unrelated subset n = 9; Supplementary Table S3. Overall, excluding these individuals yielded only the expected, minor shifts in inbreeding and relatedness metrics without altering any core conclusions. In the unrelated subset, the mean total length of runs of homozygosity (ROH) per individual decreased by 6.5% (from 348.5 Mb to 326.0 Mb), which corresponded with a modest 4–6% reduction across the mean inbreeding coefficients (F_ROH_, F_HOM_, F_UNI_, F_GRM_). Genome-wide genetic diversity remained highly stable; expected heterozygosity H_e_ was virtually identical (0.3349 vs. 0.3347), observed heterozygosity H_o_ decreased marginally by 2.8%, and nucleotide diversity (π) showed a minimal difference of 1.8% (7.12 × 10^−4^ vs. 7.25 × 10^−4^). The trajectory of the LD-based effective population size N_e_ maintained its distinct historical decline, with recent N_e_ estimates (~50 generations ago) showing a consistent downward shift in N_e_ estimates at all time depths: at ~5 generations ago, the n = 9 estimate (N_e_ ≈ 122) is lower than n = 11 (N_e_ ≈ 160); at ~10 generations ago, n = 9 gives Ne ≈ 304 vs. N_e_ ≈ 423 in n = 11; at ~50 generations ago, N_e_ ≈ 1247 (n = 9) vs. 1761 (n = 11); and at ~100 generations ago, N_e_ ≈ 3629 (n = 9) vs. 3865 (n = 11). The directional trajectory of sustained historical decline is fully preserved in the n = 9 dataset, confirming the robustness of the N_e_ trend to the presence of related individuals. N_e_ estimation was not possible for intermediate time depths (~100–400 generations ago) in the n = 9 dataset, as background LD at those distances approached the sample-size correction threshold (1/n = 0.111), yielding near-zero corrected r^2^ values and unreliable N_e_ estimates.

## 3. Discussion

### 3.1. Population Context and Sampling Strategy

Before interpreting the genomic results, it is important to contextualise the sampled population within the broader demographic landscape. According to the 20th Livestock Census [23], the total mithun population across India stands at approximately 384,000 individuals, with Arunachal Pradesh holding the largest share (~70%), followed by Nagaland, Manipur, and Mizoram. The 11 individuals sequenced in the present study were drawn from five geographically distinct villages across two districts of Arunachal Pradesh (East Siang and Lepa Rada), with the aim of maximising spatial coverage and thus the probability of capturing inter-herd genetic variation. Animals were selected as phenotypically unrelated adults maintained under semi-intensive conditions, with one to three individuals sampled per village to represent local herd diversity. While this sampling strategy captures geographic spread across a representative portion of the Arunachali range, the sample size of n = 11 necessarily limits the resolution of fine-scale population structure and rare variant discovery, as discussed in Section 3.8.

### 3.2. Genetic Diversity and Heterozygosity

The overall genome-wide observed heterozygosity averaged at 0.2854 and the expected heterozygosity averaged at 0.3347, together with a nucleotide diversity (π) of 7.16 × 10^−4^, indicate that Arunachali mithun harbours considerably lower genetic diversity than most commercial bovine breeds, consistent with its small effective population size and geographic isolation. For instance, commercial European taurine breeds such as Original Braunvieh (~1.6 × 10^−3^) [24], Yakutian cattle (~1.7 × 10^−3^), and Finncattle (~1.5 × 10^−3^) [22] exhibit considerably higher WGS-based π values. The reduced diversity in the Arunachali mithun relative to commercial breeds is at least partly explained by the founder effect of domestication from a small number of wild gaur parents, its geographical isolation to a montane forest habitat, and the very small effective population sizes which have been maintained by traditional husbandry for centuries [3,6]. The negative mean H_o_ − H_e_ value (−0.0493) also support a genome-wide heterozygosity deficit mainly caused by inbreeding and population substructure [8].

### 3.3. Pairwise Relatedness and Population Substructure

The pairwise allele sharing analysis revealed that individuals 11 and 6 share 95.3% of their alleles genome-wide, far exceeding the background inter-individual sharing of ~67–70% indicating probable first-degree kinship (full-sibling or parent-offspring relationship). Similarly, individuals 1, 2, and 3 formed a sub-cluster with 77–91.5% allele sharing, consistent with second-degree relatedness. The presence of closely related individuals within this small sample has several practical implications. First, it reduces the effective number of independent genomes contributing to population-level estimates, potentially upwardly biasing inbreeding coefficients (F_HOM_, F_GRM_) and inflating short-range LD. Second, and more importantly from a conservation standpoint, it indicates that close-relative matings are occurring within village herds without any awareness or management intervention, since no pedigree records exist for these animals. The clustering of related individuals by geographic locality suggests that isolated village herds are breeding within themselves, accelerating inbreeding at the local level even if the broader mithun population retains some diversity. This underscores the practical recommendation that even a minimal genomic kinship screening using the pairwise distance matrix generated here could serve as a first-line tool for identifying and discouraging close-relative pairings in village-level breeding decisions.

### 3.4. Inbreeding Coefficients and Runs of Homozygosity

The agreement between all four inbreeding coefficients (F_ROH_ = 0.1445, F_HOM_ = 0.1473, F_UNI_ = 0.1432, F_GRM_ = 0.1432) around 0.143–0.147 increases trust in the inbreeding estimates, since each technique reflects a different facet of autozygosity [17,18]. The strong correlation between F_UNI_ and F_GRM_ (r = 0.89) is expected given that both rely on the genomic relationship matrix [17], whereas F_ROH_’s low correlation with the others (r = 0.05–0.27) reflects its sensitivity to the ROH minimum length threshold and its specific sensitivity to runs of autozygosity arising from the co-inheritance of long haplotype tracts, rather than population-level frequency deviations. An F_ROH_ of approximately 0.14 corresponds to an effective inbreeding equivalent to that expected from the mating of second-degree relatives over several generations, a level that, while moderate, is sufficient to raise conservation concern [9].

The ROH length class distribution reveals that the inbreeding burden in Arunachali mithun is mostly of very old origin [10]. The very high proportion of short ROH (100–250 kb; 93.2%) compared to long segments is in agreement with the disappearance of extended autozygous segments over the generations through recombinations; in other words, it reflects inbreeding that took place many tens to even a hundred generations ago [9].

### 3.5. Linkage Disequilibrium and Effective Population Size

The LD decay pattern shows r^2^ falling from ~0.57 to a baseline of ~0.33 over 4–5 Mb, which means that LD covers significantly larger genomic regions in Arunachali mithun than in commercial taurine breeds (in which r^2^ usually decays to the baseline within 100–200 kb) [3,25]. This elongated LD is a manifestation of the small N_e_ of the population: fewer effective individuals mean fewer recombination events occurring between chromosomes from one generation to another, which in turn leads to the persistence of LD over longer distances [20]. The LD decay profile observed here is consistent with the N_e_ estimates and the overall diversity levels, and it has practical implications for genomic-based association studies and genomic selection programmes [3], where denser marker panels may be required to tag all functional variants given the extended haplotype structure (Supplementary Figure S5).

The Ne trajectory reveals a sustained contraction from approximately 101,850 (~2228 generations ago) to ~160 (~5 generations ago), consistent with domestication bottlenecks and geographic isolation. The n = 9 sensitivity analysis yielded directionally identical but consistently lower estimates across all time depths, confirming the robustness of the trend to relatedness-inflated LD. These estimates should be treated as order-of-magnitude indicators given the small sample size; even as upper bounds, a recent N_e_ of ~160 warrants urgent conservation attention, as it approaches the minimum viable population threshold of Ne ≥ 50–100 [3,26]. The dual-dataset approach utilising thinned (unpruned) data for LD decay characterisation and LD-pruned data for N_e_ estimation is standard practice and is justified in Section 4.12 [21,27].

### 3.6. Conservation Implications and Management Recommendations

From a conservation management perspective, the genomic data presented here also highlight three critical operational gaps in the current management of the Arunachali mithun. First, no system of individual identification exists for animals in village herds, making it impossible to track animals across time or between herds. Ear-tagging or biometric identification, as a minimum standard, would be a prerequisite for any structured breeding programme. Second, parentage verification now achievable cost-effectively using a low-density SNP panel derived from this study’s data is essential to confirm or rule out the close-relative pairings inferred here from genomic distance alone, and to prevent further inadvertent inbreeding. Third, performance testing for economically important traits (body weight, growth rate, disease resistance) is entirely absent in this population. Without phenotypic records linked to genotyped individuals, genome-wide association studies and genomic selection, the logical next steps from this genomic baseline, cannot be implemented. Establishing even a minimal performance recording system in parallel with expanded genomic sampling would substantially increase the translational value of future studies [3,6].

### 3.7. Power Analysis and Sample-Size Considerations

A retrospective power assessment is warranted to clarify which conclusions from this study are well-supported by n = 11 and which require cautious interpretation pending expanded sampling. For estimates of genomic parameters that are functions of heterozygosity (H_o_, H_e_, π), analytical and simulation studies indicate that as few as 5–10 diploid individuals provide nearly unbiased estimates of nucleotide diversity (π) when sequencing depth is sufficient and the loci are numerous [28]. This is because π is an estimator averaged across millions of SNPs, and its standard error scales with 1/√L (where L is the number of windows or loci), not with sample size alone. With L ≈ 48,000 windows retained in this study, the SE of our π estimate (2.21 × 10^−6^) is indeed very small relative to the mean (7.16 × 10^−4^), indicating that π is estimated with high precision despite the small n. The same logic applies to the genome-wide mean inbreeding coefficients: F_ROH_, F_HOM_, F_UNI_, and F_GRM_ are all averages across large numbers of loci, and their tight convergence around 0.143–0.147 across methodologically independent estimators provides internal cross-validation that is robust to n.

By contrast, LD-based N_e_ estimation is known to be sensitive to sample size. The standard Waples [21] correction for sampling noise in r^2^ is −1/n per locus pair, which at n = 11 equals approximately −0.091. At the short inter-SNP distances where our raw r^2^ values are highest (≈0.57), this correction is relatively small and the N_e_ estimates are therefore reasonably stable. At longer distances where r^2^ approaches the plateau (≈0.33), the proportional correction is larger and N_e_ estimates carry greater uncertainty. Using the formula of Waples and Do [29] the 95% confidence interval around our most recent N_e_ estimate of ≈160 spans roughly ±40–80 individuals given n = 11. While this interval is wide in absolute terms, it does not encompass zero and does not overlap with any N_e_ value above ≈400, reinforcing the directional conclusion that recent N_e_ is substantially lower than the ancestral N_e_.

Taken together, this power assessment suggests that the headline diversity metrics (π, H_o_, H_e_, mean F_ROH_, LD decay profile) are the most reliable outputs of the present study and can be treated as provisional species-level benchmarks. The N_e_ trajectory and PCA-based substructure patterns are directionally informative but carry wider uncertainty bounds. All other metrics that depend on allele frequency estimation at low counts, particularly the MAF-distribution shape and HWE deviation statistics, should be interpreted as exploratory and replicated with larger samples before forming the basis of management decisions.

### 3.8. Population Genetic Structure

All individuals were assigned to a single ancestral cluster (Q = 1.000 at K = 1), and at K = 2 through K = 4 the apparent cluster assignments are inconsistent across K values and do not resolve discrete groupings, indicating that the visual separations observed at higher K reflect sampling noise and the relatedness signal from the pairs 6/11 and 1/2/3 rather than true population structure. This finding is biologically consistent with the semi-domesticated management system of mithun, in which animals range freely across village territories and are not subject to structured pedigree-based breeding that would generate reproductive isolation sufficient to produce discrete ancestral clusters. The K = 1 result should, however, be interpreted with the caveat that the sample size (n = 11) limits the statistical power to detect subtle admixture gradients or fine-scale substructure; expanded sampling across geographic localities is required before population panmixia can be concluded definitively.

### 3.9. Sensitivity Analysis: Effect of Relatedness on Population Parameters

To assess the robustness of our key results to this source of bias, all principal population genetic analyses were repeated on an unrelated subset of n = 9 individuals (1, 2, 4, 5, 6, 7, 8, 9, 10), generated by excluding one individual from each related pair (3 and 11 removed). Full results of this sensitivity analysis are provided in Supplementary Table S3. In brief: (i) the mean inbreeding coefficients (F_ROH_, F_HOM_, F_UNI_, F_GRM_) decreased by 4–6% in the unrelated subset, consistent with the expected removal of the relatedness-driven homozygosity inflation; (ii) the nucleotide diversity (π) and heterozygosity (H_o_, H_e_) were virtually unchanged (≤2% difference); (iii) the LD-based Ne trajectory retained its directional decline toward the present, with a modest upward shift (~7%) in recent Ne estimates, consistent with the known upward inflation of LD-based N_e_ by related samples; and (iv) the ADMIXTURE result remained K = 1. These results confirm that the headline conclusions of this study are robust to the presence of related individuals. The full n = 11 dataset is retained for the primary analysis to maximise the statistical power for genome-wide metrics, while the unrelated-subset results serve as a robustness check.

### 3.10. Comparison with Published Bovid Datasets

To contextualise the genetic diversity metrics reported here, Table 2 places the Arunachali mithun results alongside published WGS and high-density SNP array-derived estimates from a range of bovid species and breeds, spanning commercial taurine breeds, indigenous zebu cattle, wild bovids, and prior mithun studies. The Arunachali mithun occupies a distinct position in this diversity landscape: the nucleotide diversity (π = 7.16 × 10^−4^) is substantially lower than that of all commercial taurine breeds and most indigenous zebu populations, but is broadly comparable to other populations with documented small effective sizes and geographic isolation. The convergence of all four inbreeding estimators around 0.143–0.147 and the predominance of short ROH (<250 kb) together place the Arunachali mithun closest to the ancient-inbreeding profile documented in the Yunnan gayal [2] and Indian mithun SNP-array data [3], while the LD-decay baseline of r^2^ ≈ 0.33 at >4 Mb is notably more extended than that of any commercial taurine breed, consistent with the reduced N_e_ estimated here.

### 3.11. Study Limitations

Several limitations of the present study must be acknowledged explicitly. First, the sample size of n = 11 is the most consequential constraint. While WGS data provide millions of informative loci, many population-level statistics, including minor allele frequency spectra, Hardy–Weinberg equilibrium tests, LD-based effective population size estimation, and population structure inference are sensitive to the number of sampled individuals rather than the number of genotyped loci. With 11 diploid individuals, the maximum minor allele count is 22, meaning MAF classes below approximately 1/22 ≈ 0.045 are entirely unobservable. This produces the discrete, quantized MAF distribution reported here and limits the resolution of the low-frequency end of the allele frequency spectrum. Rare variants, which carry disproportionate information about recent demographic events and population-specific adaptation, are therefore systematically underrepresented. Similarly, the power to detect subtle population substructure or to partition variance among geographic sampling localities is substantially reduced relative to studies with n ≥ 30–50.

Second, the moderate sequencing depth of approximately 10× per individual, while sufficient for SNP calling in a joint-genotyping framework, reduces genotyping accuracy at heterozygous sites relative to higher-coverage protocols. Heterozygote under-calling at low depth can inflate apparent homozygosity, leading to an upward bias in inbreeding coefficient estimates (F_HOM_, F_GRM_) and an increase in the number of ROH segments detected, particularly short segments near the minimum length threshold. The observed heterozygosity deficit (H_o_ − H_e_ = −0.0493) may therefore partly reflect depth-related genotyping error rather than true biological inbreeding alone; this cannot be fully disentangled with the present data.

Third, sequencing was conducted in two batches at two service providers using different library preparation kits (KAPA Hyper Plus and Illumina TruSeq Nano), introducing a potential batch effect. Although the GATK joint-genotyping pipeline partially mitigates kit-specific biases by normalising depth and applying uniform hard-filter thresholds across all samples, residual inter-batch differences in adapter ligation efficiency, GC-content bias, and insert-size distribution cannot be fully excluded. The principal component analysis (PCA) did not reveal a clean batch-driven separation, suggesting the effect is not dominant, but a formal permutation test for batch effects was not performed and should be considered in any re-analysis with expanded sampling.

Fourth, the reference genome used here, the *Bos frontalis* YUN_Gayal_1 assembly (GCA_043643345.1), represents a single Yunnan gayal individual and may not perfectly reflect the Arunachali mithun haplotype architecture. Reference bias, the systematic underrepresentation of alleles that differ from the reference haplotype, can suppress heterozygosity calls at divergent loci. The use of a graph-based or pan-genome reference in future work would mitigate this limitation. Fifth, the complete absence of pedigree records for the sampled individuals prevents the direct validation of genomic inbreeding coefficients against pedigree-derived estimates (F-pedigree), a step that is standard practice in livestock genomics. The kinship signals detected here through PCA and pairwise distance metrics (e.g., 6–11, 1–3 sub-clustering) are suggestive of close relatedness, but their precise genealogical basis cannot be ascertained from these data alone.

## 4. Materials and Methods

### 4.1. Sample Collection and Sequencing

A total of 11 Arunachali mithun individuals (1–11) kept under a semi-intensive rearing system were selected for blood sampling in Siang region of Arunachal Pradesh, India. The eleven individuals representing distinct geographic localities were obtained, with blood samples collected from five villages across the Lepa Rada and East Siang districts of Arunachal Pradesh: Riga village (East Siang district), Yibuk village (East Siang district), Basar village (Lepa Rada district), Sago village (Lepa Rada district), Echi Chiku (Lepa Rada district), and Echi Sik (Lepa Rada district). The geographic coordinates of all sampling sites are provided in Table 3. Two ml of blood was drawn from the jugular vein. All animal sampling procedures were conducted in compliance with institutional guidelines and approved under the ICAR-NRC Mithun Institutional Animal Ethics Committee (IAEC) protocols (approval F.No. NRCM/IAEC/2023(08)/30). Genomic DNA was extracted from peripheral blood using the Wizard Genomic DNA Purification Kit (Promega Corporation, Madison, WI, USA) following the manufacturer’s protocol, quantified by NanoDrop spectrophotometry (Thermo Fisher Scientific, Waltham, MA, USA), and assessed for integrity by 2% agarose gel electrophoresis. Whole-genome paired-end sequencing libraries were prepared across two sequencing batches: five samples were processed with the KAPA Hyper Plus Kit (Roche Diagnostics, Basel, Switzerland) and the remaining six samples were processed with the Illumina TruSeq Nano DNA Library Prep Kit (Illumina, Inc., San Diego, CA, USA). DNA was fragmented to a mean insert size of approximately 275–380 bp, end-repaired, A-tailed and ligated with indexed adapters. Libraries were size-selected with AMPure XP beads (Beckman Coulter, Brea, CA, USA), validated on an Agilent TapeStation (Agilent Technologies, Inc., Santa Clara, CA, USA), and sequenced on an Illumina platform with 2 × 150 bp paired-end chemistry to a target yield of ~30 Gb per sample (~10× average autosomal coverage). Raw paired-end reads were processed with fastp v0.20.1 [35] using paired-end adapter auto-detection (--detect_adapter_for_pe) and bidirectional sliding-window quality trimming (--cut_front--cut_right, 4 bp window, mean quality threshold Q20); reads shorter than 30 bp after trimming were discarded.

### 4.2. Read Alignment and Variant Calling

Quality-trimmed reads were aligned to the *Bos frontalis* YUN_Gayal_1 reference genome (GCA_043643345.1) using BWA-MEM (version 0.7.17) with the −M flag and per-sample read group assignment [36]. The resulting SAM files were coordinate-sorted with SAMtools 1.23.1 [37]. PCR duplicate marking was performed using GATK 4.5 [38]. MarkDuplicates with an optical duplicate pixel distance of 2500 (appropriate for patterned flow cells) [14]. Individual gVCF files were generated using GATK HaplotypeCaller in GVCF emission mode with a minimum genotype quality of 30 and paired-HMM AVX implementation for computational efficiency. Joint genotyping was performed across all individuals using GATK CombineGVCFs and GenotypeGVCFs, with heterozygosity priors of 0.005 and indel heterozygosity of 0.001 [14].

### 4.3. Variant Filtration and Quality Control

SNPs were extracted from the joint VCF using GATK SelectVariants and subjected to hard-filtering with the following GATK VariantFiltration thresholds: QD < 2.0, QUAL < 30.0, SOR > 3.0, FS > 60.0, MQ < 40.0, MQRankSum < −12.5, and ReadPosRankSum < −8.0 [14]. Only autosomal chromosomes were retained. Following GATK hard filtering, variants were further filtered using BCFtools 1.18 [37] to remove sites with extreme depth of coverage (DP < 5 or DP > 100; bcftools filter −e “INFO/DP < 5 || INFO/DP > 100”) and to exclude singleton variants (allele count = 1; bcftools view −e ‘AC = = 1’), as singletons in small sample sizes are unreliable and disproportionately inflate rare allele estimates. The depth filter removes poorly covered sites likely to harbour genotyping errors and high-coverage sites that may represent collapsed repeats or paralogous regions. Additional population-level quality control was applied in PLINK (v1.9) [15,39] with --chr-set 28, filtering for minor allele frequency (MAF ≥ 0.05), Hardy–Weinberg equilibrium (*p* ≥ 1 × 10^−5^), and genotype missingness (<5%). The final QC-passed dataset comprised 4,943,593 high-quality biallelic SNPs across 11 samples (Table 4). SNPs were annotated with custom identifiers (CHROM_POS_REF_ALT) using bcftools annotate. The MAF ≥ 0.05 threshold was applied to remove low-frequency variants whose allele frequency estimates are unreliable given the limited sample of 22 alleles (n = 11), where individual genotyping errors can substantially distort allele counts at very low frequencies. This conservative filter is appropriate for heterozygosity, LD, ROH, and Ne analyses in small-sample WGS datasets, as low-frequency variants inflate noise in pairwise LD estimation and generate spurious short ROH segments. The trade-off is the exclusion of rare variants (AC = 2–3), which may cause a modest underestimation of nucleotide diversity relative to unfiltered estimates; this limitation is explicitly acknowledged in Section 3.11.

### 4.4. Hardy–Weinberg Equilibrium and Heterozygosity

The HWE tests and observed/expected heterozygosity were computed genome-wide using PLINK--hardy (--chr-set 28) [15]. Observed heterozygosity (H_o_) and expected heterozygosity (H_e_) were extracted from the PLINK.hwe output file. Chromosome-level mean H_o_ and H_e_ were calculated in R (v4.3) [40] using dplyr. *p*-values were corrected for multiple testing using both FDR (Benjamini–Hochberg) and Bonferroni methods. The genome-wide mean, standard deviation (SD), and standard error (SE) of H_o_, H_e_, and their difference (H_o_ − H_e_) were computed. The HWE *p*-value distribution was visualised using a histogram, and the observed versus expected heterozygosity was plotted as a scatter plot with a 1:1 reference line.

### 4.5. Minor Allele Frequency Distribution

MAF was estimated across all QC-passed SNPs using PLINK--freq [15]. The distribution was visualised as a histogram with an overlaid kernel density curve, binned at intervals of 0.05 across the MAF range of 0.05–0.50. This threshold was applied to remove variants with unreliable frequency estimates at n = 11 (minimum allele count = 1/22 chromosomes ≈ MAF 0.045); note that this excludes rare variants and may modestly upwardly bias diversity estimates and ROH detection.

### 4.6. Nucleotide Diversity

Genome-wide nucleotide diversity (π) was estimated using VCFtools (v0.1.16) [16] with a sliding window size of 50 kb (--window-pi 50,000). Windows with fewer than 20 variant sites were excluded from the analysis. Summary statistics (mean, SD, SE) were calculated for both the genome-wide dataset and on a per-chromosome basis. The genomic distribution of π was visualised as a faceted line plot across all autosomes.

### 4.7. Principal Component Analysis

Principal component analysis (PCA) was conducted using PLINK V2.0 alpha [15,41] (--pca 10--chr-set 28) on the full LD-pruned SNP dataset (--indep-pairwise 50 5 0.2) to visualise genomic relationships among the 11 individuals in a model-free framework. Principal component analysis (PCA) captures the directions of maximum genotype variance and complements the pairwise distance analysis by revealing continuous population structure, differentiation, and potential outliers without an underlying population model. The first two principal components (PC1 and PC2) were plotted using ggplot2 in R. The amount of variance explained by each PC was calculated. PCA was combined with the pairwise genetic distance heatmap to corroborate patterns of substructure, relatedness, and possible duplicate sampling.

### 4.8. Pairwise Genetic Distance and Allele Sharing

Average pairwise genetic distance (D) was estimated from IBS (identity-by-state) scores computed using PLINK--distance ibs flat-missing [15]. The full distance matrix was constructed and D was calculated as 1 − IBS for all pairwise comparisons. The allele sharing (D_ST_ = 1 − D) was also computed. Summary statistics (mean D, SD, SE) were derived from the upper triangle of the distance matrix. Hierarchically clustered heatmaps of both D and D_ST_ were generated using pheatmap in R.

### 4.9. Inbreeding Coefficients

Four complementary genomic inbreeding coefficients were estimated [18]. F_ROH_ was derived from runs of homozygosity detected by detectRUNS [42] (see Section 4.10) as the ratio of the total ROH length to the autosomal genome length (2,436,710,000 bp). F_HOM_ was computed using PLINK--het [15], which estimates the proportion of excess homozygosity as [O(HOM) − E(HOM)]/[N(NM) − E(HOM)]. F_UNI_ (GCTA Fhat3) was computed as the correlation between uniting gametes using the genomic inbreeding coefficient module of GCTA (--ibc, --autosome-num 28) [17]. F_GRM_ was derived from the diagonal of the genomic relationship matrix (GRM) computed by GCTA (--make-grm) as diagonal (GRM) − 1 [17]. Pearson correlations among the four coefficients were computed and visualised using ggcorrplot.

### 4.10. Runs of Homozygosity

ROH were detected using the detectRUNS 0.9.6 package in R (slidingRUNS.run function) with the followingparameters: window size = 25 SNPs, heterozygosity threshold = 0.05, minimum SNP = 25, maximum heterozygous SNPs per window = 0, maximum missing SNPs per window = 1, maximum gap = 100,000 bp, and minimum ROH length = 100,000 bp (100 kb) [18].ROH segments were classified into length bins (100–250, 250–400, 400–550, 550–700, 700–1000 kb) to distinguish between ancient, moderate, and recent inbreeding contributions [9,10,43]. A genome-wide Manhattan plot of the ROH segment positions and lengths was generated. The 100 kb minimum ROH length was chosen to be consistent with the detectRUNS default and with WGS-based ROH studies in bovids that include short autozygous segments reflecting ancient population history [11]. This threshold is lower than the ≥1 Mb threshold commonly used in array-based livestock studies, and it is appropriate for WGS data where short segments can be reliably distinguished from genotyping noise given the high marker density. However, the use of 100 kb as the minimum threshold unavoidably inflates the count of ‘ancient’ ROH and the FROH estimate relative to studies using higher thresholds; this limitation is acknowledged in the Results (Section 2.9) and Discussion (Section 3.4), and the ROH-based inbreeding conclusions are explicitly qualified as reflecting detectable ancient inbreeding under this threshold.

### 4.11. Linkage Disequilibrium and Haplotype Block Analysis

LD between all SNP pairs was computed per chromosome using PLINK--r2 with parameters--ld-window-kb 5000 and --ld-window-r2 0 on LD-thinned data [15]. Thinning was performed using mapthin [44] (−b 20). LD summary statistics were computed by binning pairwise comparisons into distance intervals (0–1 kb, 1–5 kb, 5–10 kb, 10–50 kb, 50–100 kb, 100–500 kb, 500 kb^−1^ Mb, >1 Mb). The genome-wide LD decay was plotted as r^2^ against physical distance. Haplotype block structure was inferred using PLINK--blocks (--no-pheno-req, --chr-set 28). The block size distribution was visualised on a log-scale histogram (Supplementary Figure S5 and S6). This analysis used the full set of quality-controlled, thinned SNPs without LD pruning, as LD decay characterisation requires the retention of correlated SNP pairs to accurately estimate the decline in r^2^ with physical distance. This analytical dataset is distinct from the LD-pruned dataset used for N_e_ estimation, the rationale for using different datasets for these two analyses is provided in Section 4.12.

### 4.12. Effective Population Size Estimation

The N_e_ estimation requires a different analytical dataset from the LD decay characterisation in Section 4.11. Whereas the LD decay analysis retains correlated SNP pairs to measure the rate of r^2^ decline with physical distance, N_e_ estimation using the Hill [45] formula requires that the mean r^2^ per distance bin reflects genuine residual LD above the background sampling noise floor. If highly correlated SNP pairs are retained, short-distance bins will be dominated by the LD signal itself rather than the per-pair r^2^ values that are informative for N_e_. Accordingly, Ne was estimated from a separately LD-pruned dataset (n11_pruned_50k; PLINK--indep-pairwise 200 25 0.3, retaining 19,222 SNPs after pruning at r^2^ < 0.3 within 200-SNP sliding windows), which removes redundant correlated pairs while retaining representative SNP coverage across all chromosomal distance bins. After applying the Waples [21] sample-size correction (subtracting 1/n from the mean r^2^ per bin), the small residual corrected r^2^ values (0.007–0.016 per bin) represent the genuine LD signal above the noise floor and are the direct inputs to the N_e_ formula. This dual-dataset approach, utilising thinned (unpruned) data for LD decay characterisation and LD-pruned data for N_e_ estimation, is standard practice in population genomics and ensures that each analysis is conducted on the dataset most appropriate for its statistical requirements [21,27,46,47]. 

Historical effective population size (N_e_) was estimated from linkage disequilibrium data derived from PLINK (--r2, --ld-window-kb 10,000, --ld-window-r2 0) on the LD-pruned dataset (n11_pruned_50k; PLINK--indep-pairwise 200 25 0.3, 19,222 SNPs). The same pruned dataset was used for both the full n = 11 analysis and the n = 9 sensitivity analysis, with the only difference being the exclusion of individuals AP3 and AP11 in the latter via a --keep file. SNP pairs on the same chromosome within a 10 Mb window were retained. Pairs with r^2^ = 0 or r^2^ = 1 were excluded. The remaining pairs were binned by inter-marker physical distance in 50 kb intervals, and the mean r^2^ was calculated per bin. The N_e_ was estimated per bin using the formula: N_e_ = (1/(4c)) × ((1/r^2^) − 1), where c is the recombination rate in Morgans estimated assuming 1 cM/Mb (c = distance in bp × 10^−8^), and the corresponding time depth per bin was calculated as t = 1/(2c) generations [21,27]. The Waples [21] sample-size correction (r^2^_corrected = mean r^2^ − 1/n) was applied to all bins. Bins where r^2^_corrected fell at or below 0.005 were excluded as near-zero values produce numerically unstable N_e_ estimates; this resulted in 15 excluded bins for n = 11 (first excluded bin: midpoint 75 kb, ~670 generations ago) and 11 excluded bins for n = 9 (first excluded bin: midpoint 125 kb, ~400 generations ago), corresponding to intermediate time depths where background LD in the pruned dataset approached the correction threshold. The [0, 50] kb bin is partially affected by the 50 kb pruning window, and the ancient N_e_ estimate (~101,850) should be treated as indicative rather than precise. N_e_ estimates at 5–100 generations ago (distance bins > 800 kb, outside the pruning window) are the most robust outputs of this analysis. All binning and N_e_ computation were performed using a custom R script.

## 5. Conclusions

Across a high-quality SNP dataset of 4,943,593 loci, we document moderate genetic diversity (π = 7.16 × 10^−4^, H_e_ = 0.3347), a consistent heterozygosity deficit (H_o_ − H_e_ = −0.0493), and moderate inbreeding (F-coefficients ≈ 0.143–0.147) of predominantly ancient or distant origin under current detection thresholds (dominance of short ROH < 250 kb), though recent inbreeding below the 100 kb detection limit cannot be excluded given methodological constraints and small sample size. We also report a sustained historical population contraction (N_e_ declining from ~100,000 (~2000 generations ago) to ~160 (~5 generations ago), and extended LD relative to commercial taurine breeds (Hill and Weir, 1988). These genomic features collectively point to a population shaped by long-term isolation, small effective population size, and traditional non-intensive husbandry practices. The findings underscore the importance of implementing evidence-based conservation strategies for this culturally significant indigenous bovine, including pedigree-informed mating to limit the further erosion of genetic diversity. The genomic baseline established herein provides an essential foundation for future population genomic, association, and selection studies in the Arunachali mithun (*Bos frontalis*).

## Figures and Tables

**Table 1:** Distribution of ROH segments by length class in Arunachali mithun.

ROH Length Class	N Segments	% of Total	Interpretation	Ancestry
100–250 kb	23,186	93.2%	Very Short	Ancient
250–400 kb	1551	6.2%	Very Short	Ancient
400–550 kb	175	0.7%	Very Short	Ancient
550–700 kb	19	0.08%	Short	Distant
700–1000 kb	6	0.02%	Short	Distant
Total	24,937	100%	-	-

**Table 2:** Comparison of headline genetic diversity metrics between Arunachali mithun and published bovid datasets.

Species/Breed	n	Data Type	π (×10^−4^)	H_o_	H_e_	Mean F_ROH_	N_e_ (Recent)	Reference
**Arunachali mithun (*Bos frontalis*)**	**11**	**WGS (~10×)**	**7.16**	**0.285**	**0.335**	**0.145**	**~160**	**This Study**
**Yunnan Gayal/Mithun (*Bos frontalis*)**	1	WGS	~6.8	0.276	0.312	NR	NR	[2]
**Indian Mithun (SNP array, 8 breeds)**	166	777 K array	NA	0.293	0.309	0.078	~90–170	[3]
**Original Braunvieh (taurine)**	~50	WGS	~16	…	…	…	…	[24]
**Yakutian cattle (taurine)**	~20	WGS	~17	…	…	….	…	[22]
**Finncattle (taurine)**	~20	WGS	~15	…	…	…	…	[22]
**Qinghai yak (*Bos grunniens*)**	113	WGS	~13–18	…	…	Low; varies by breed	…	[30]
**European bison (*Bison bonasus*)**	183/8	SNP array (BovineHD)/WGS	NR (~14 divergence)	<0.30 (microsatellite)	NR	Very high (F_ROH_ ≈ 0.52–0.56)	~16–24 (pedigree)	[31,32,33]

F_ROH_ values are computed from SNP-array data and may not be directly comparable to WGS-derived F_ROH_ values due to ascertainment bias and differences in minimum ROH length thresholds applied. π values for Original Braunvieh are from Bhati et al. [24]; π values for Yakutian cattle and Finncattle are from Weldenegodguad et al. [22] π values for Qinghai yak are from Wang et al. [30]. For European bison: n = 183 refers to SNP-array genotyped individuals [32]; n = 8 refers to WGS individuals [31]. π is not directly comparable as sliding-window estimates are unavailable; the value shown (~14) reflects nucleotide divergence from the cattle reference genome [33], not intra-population π. H_o_ is from microsatellite data. F_ROH_ is from WGS data [31]. N_e_ is pedigree-based [34]. Abbreviations: NA, not applicable (the metric cannot be meaningfully computed for this data type, e.g., π from SNP-array data due to ascertainment bias); NR, not reported in the original publication; …, specific value not available from the cited source; Important comparability caveat: this table mixes WGS-derived and SNP-array-derived estimates. Array-based H_o_, H_e_, F_ROH_ and N_e_ values (e.g., [3]: 777K array) are not directly comparable to WGS-derived values due to ascertainment bias, differences in marker density, and differences in the minimum ROH length thresholds applied in each study. Comparisons should be interpreted qualitatively rather than quantitatively. Authors are encouraged to consider adding a column explicitly flagging data type (WGS vs. array) or separating the table into WGS-only and array-based sections to improve interpretability.

**Table 3:** Geographic coordinates of blood sample collection sites for Arunachali mithun (*Bos frontalis*) whole-genome sequencing study.

Village/Site	District	Latitude (°N)	Longitude (°E)	Samples
Riga Village	East Siang	28.4341	95.0427	1, 2 and 3
Yibuk Village	East Siang	28.2800	94.6890	4 and 5
Basar Village	Lepa Rada	27.9833	94.6667	7 and 8
Sago Village	Lepa Rada	27.8167	94.5833	9 and 10
Echi Chiku	Lepa Rada	27.9667	94.6333	6 and 11

**Table 4:** Summary of SNP filtering and quality control pipeline.

Filter Parameter	Threshold Applied	SNPs Retained/Removed
Minor Allele Frequency (MAF)	≥0.05	1,781,757 removed
Hardy–Weinberg Equilibrium	*p* ≥ 0.00001	0 removed
Genotype missingness	<5%	5,891,862 removed
Final QC-passed SNPs	All filters passed	4,943,593 retained
Samples retained	-	11

## Data Availability

The data presented in this study are available on request from the corresponding author (the datasets are subjected to institutional and governmental data-sharing policies).

## Supplementary Materials

The following supporting information can be downloaded at: https://www.mdpi.com/article/10.3390/ijms27135824/s1.
