# Supplementary material for: Whole-Genome Re-Sequencing Reveals Genetic Diversity and Population History of Arunachali Mithun (Bos frontalis)
Source: Int J Mol Sci. 2026 Jun 27;27(13):5824. doi: 10.3390/ijms27135824 (PMC13362323; doi:10.3390/ijms27135824)
Supplement: Supplementary file 1 [file ijms-27-05824-s001.zip › ijms-4370507-supplementary.pdf]

Whole-Genome Re-sequencing Reveals Genetic Diversity and Population History of Arunachali Mithun (*Bos frontalis*)

Chotso et al.

SUPPLEMENTARY MATERIAL

Supplementary Figures

This file contains six supplementary figures (Figures S1–S6) that support the analyses presented in the main manuscript. Specifically, these figures detail the minor allele frequency (MAF) distribution (Section 2.2); the distribution of HWE p-values and a scatter plot of observed (H_o) versus expected (H_e) heterozygosity (Section 2.3); genome-wide nucleotide diversity (Section 2.4); and haplotype block analyses (Section 2.9). Collectively, they provide additional regional and genome-wide context for the linkage disequilibrium and haplotype block structure of the Arunachali Mithun, supplementing the primary LD decay analysis shown in Figure 11. .

Figure S1.

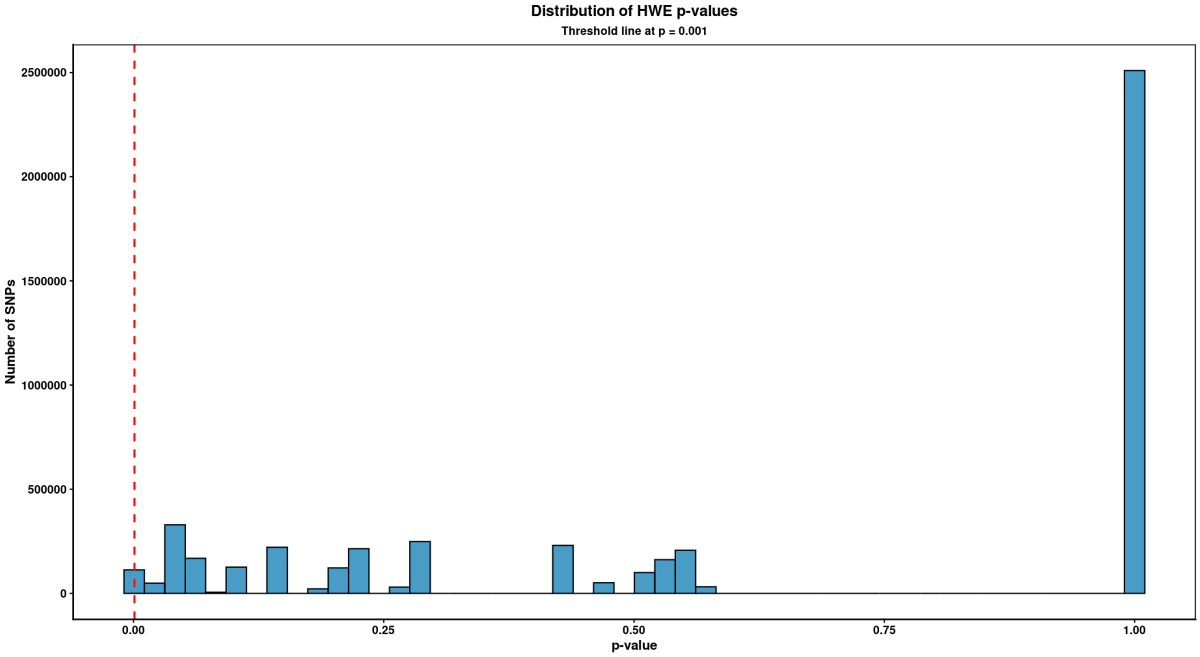

Figure S1. Distribution of HWE p-values across 4,943,593 SNPs. The red dashed line indicates the p = 0.001 threshold.

Figure S2.

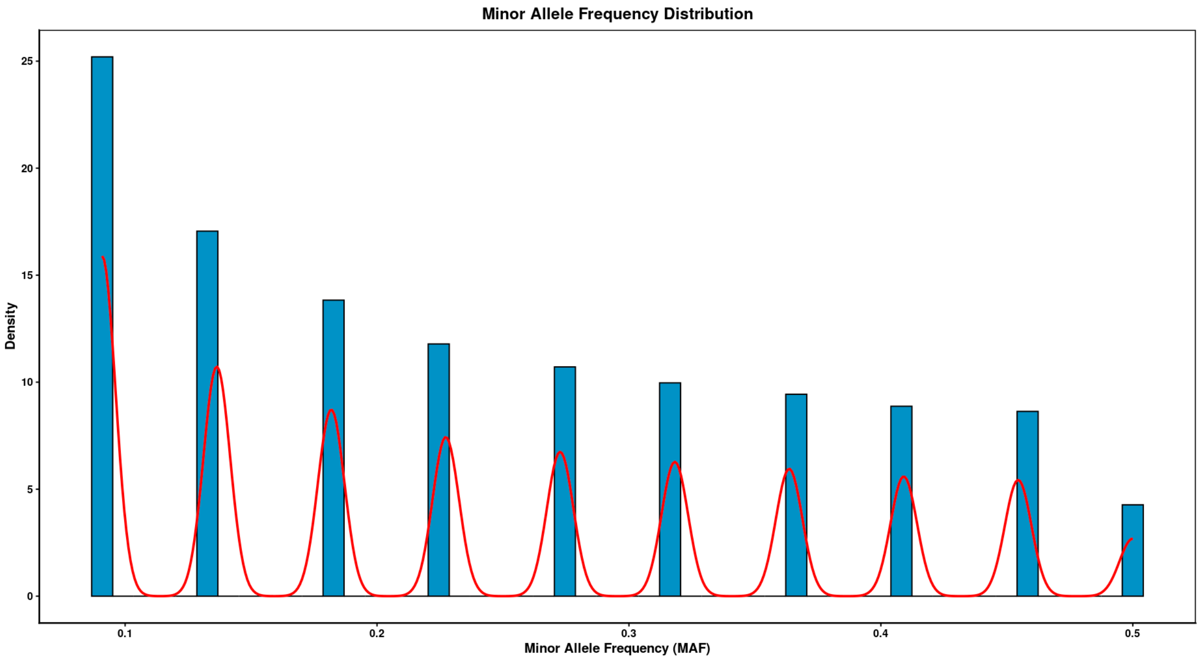

Figure S2. Minor allele frequency (MAF) distribution of 4,943,593 QC-passed SNPs in Arunachali Mithun. Blue bars represent the histogram density and the red curve shows the kernel density estimate.

Figure S3.

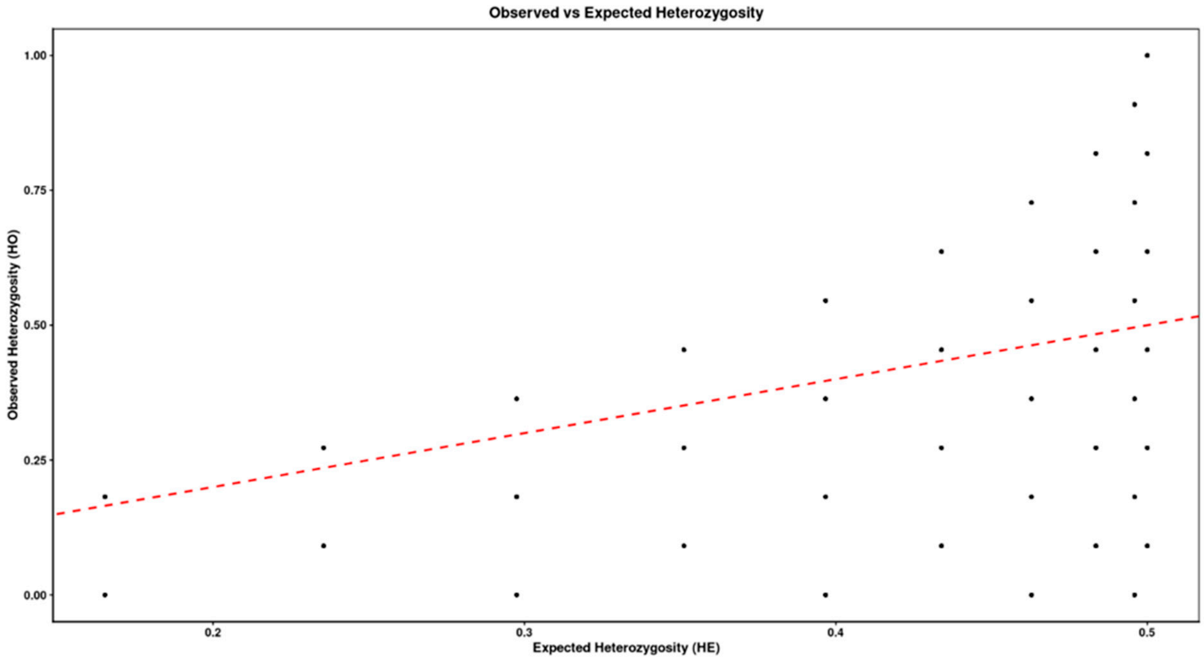

Figure S3. Scatter plot of observed (H_o) versus expected (H_e) heterozygosity per SNP. The red dashed line represents the linear regression trend.

Figure S4.

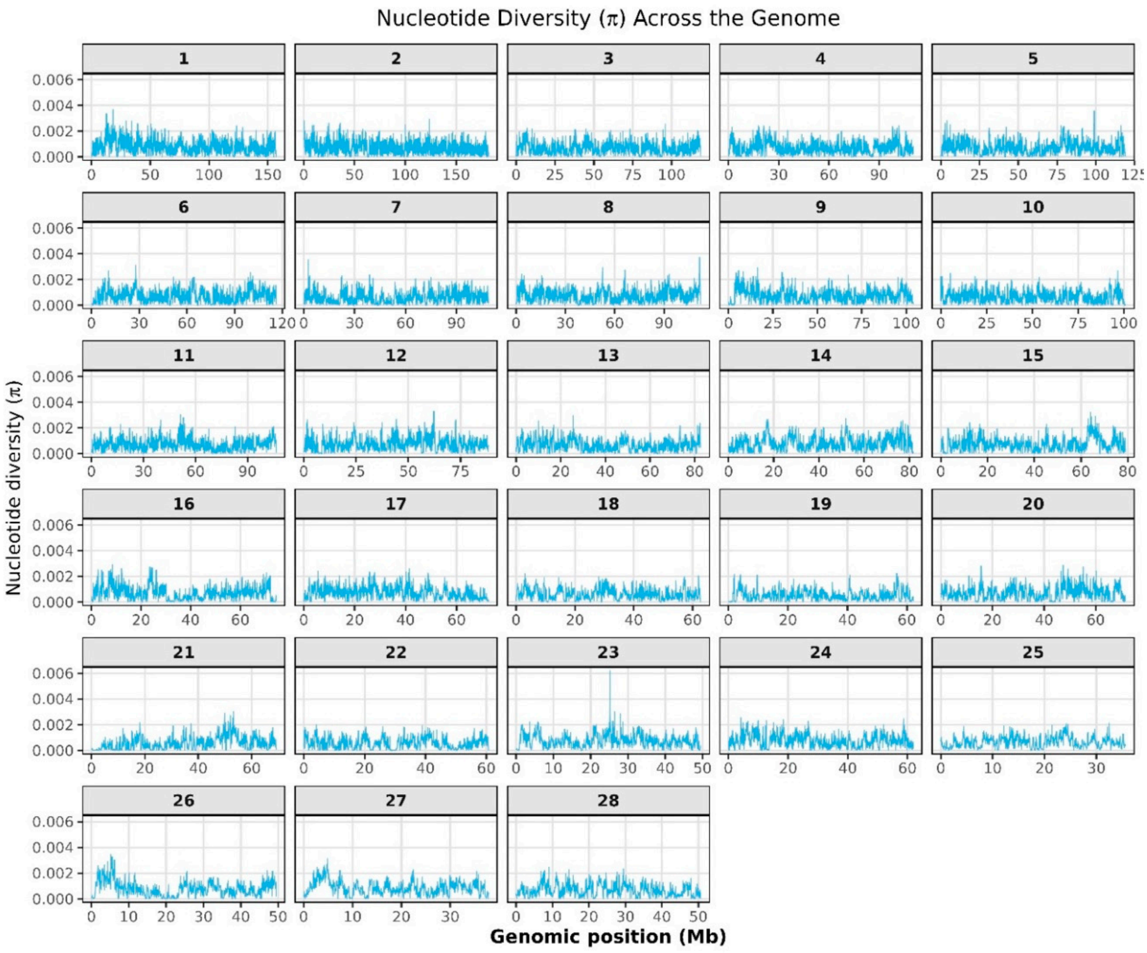

Figure S4. Genome-wide nucleotide diversity (π) estimated in 50 kb windows across all autosomes of Arunachali Mithun.

Figure S5.

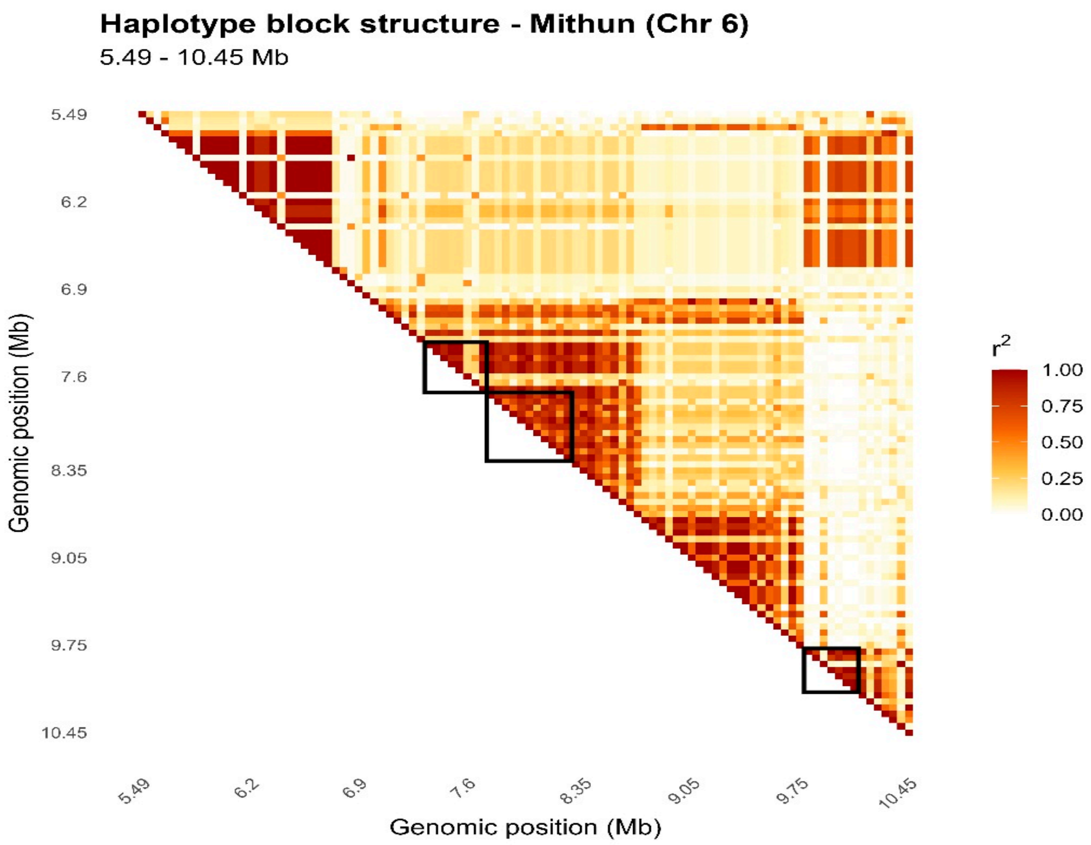

Figure S5. Haplotype block structure on chromosome 6 (5.49–10.45 Mb) of Arunachali Mithun, visualised as a triangle linkage disequilibrium (LD) heatmap. Black rectangles denote inferred haplotype block boundaries. Regions of high LD ($r^2 \approx 0.75$–1.0) are visible as dark triangular blocks, with three major haplotype blocks prominent at approximately 7.5–8.3 Mb. Haplotype blocks were inferred using PLINK --blocks; the LD heatmap was generated using R. This figure has been moved to the supplementary material given the regional and chromosome-specific nature of the analysis.

Figure S6.

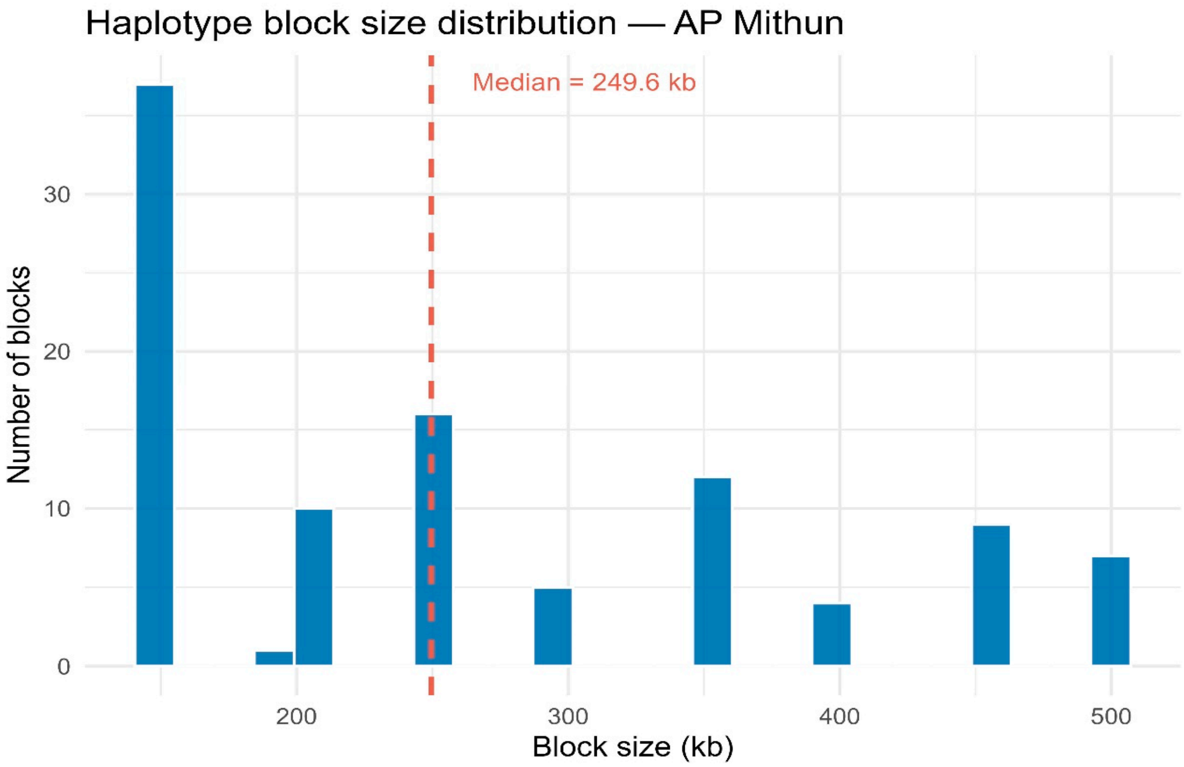

Figure S6. Distribution of haplotype block sizes (log-scale x-axis) across the Arunachali Mithun genome. The distribution shows that the most common block size is approximately 200 kb, with secondary peaks at 300 kb and 400–500 kb, consistent with the observed extended LD decay profile. Block sizes were computed using PLINK --blocks across all autosomes; the log-scale x-axis resolves the full range of block sizes from <10 kb to >1 Mb.

Figure S7

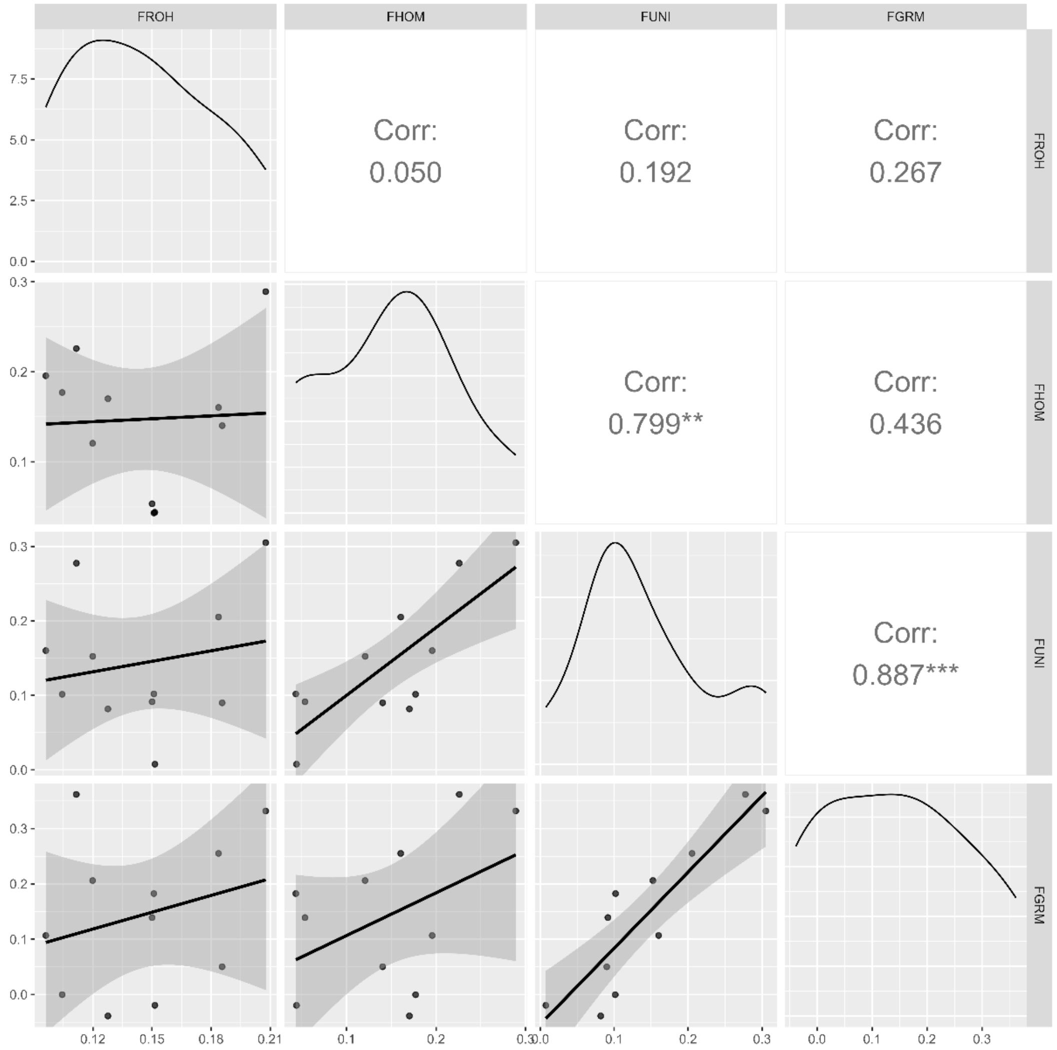

Figure S7. Pearson scatter plot matrix of pairwise relationships among the four genomic inbreeding coefficients (F_{ROH}, F_{HOM}, F_{UNI}, F_{GRM}) in 11 Arunachali Mithun individuals. Lower triangle: scatter plots with loess regression line and 95% confidence band. Diagonal: kernel density distribution of each coefficient. Upper triangle: Pearson correlation coefficients. Significance levels: $p < 0.01$, $p < 0.001$. The strong correlation between F_{UNI} and F_{GRM} (r = 0.887 reflects their shared genomic relationship matrix basis, while the low correlation of F_{ROH} with the other estimators (r = 0.050-0.267) reflects its dependence on the 100 kb minimum ROH length threshold rather than genome-wide allele frequency deviations.

Table S1.

Per-chromosome nucleotide diversity (π) in Arunachali Mithun. Values computed using VCFtools (v0.1.16) with a 50 kb sliding window (--window-pi 50000); windows with <20 variant sites excluded. Mean π, standard deviation (SD), and standard error (SE) are reported per chromosome. N Windows = number of 50 kb windows retained after filtering. The genome-wide mean π of 7.16×10^{-4} reported in the main text is the average across all retained windows.

Chromosome	Mean π	SD π	SE π	N Windows
1	0.000812	0.000515	9.19e-06	3,137
2	0.000731	0.000470	7.79e-06	3,633
3	0.000681	0.000440	9.03e-06	2,374
4	0.000714	0.000462	9.84e-06	2,203
5	0.000715	0.000468	9.60e-06	2,378
6	0.000730	0.000489	1.02e-05	2,319
7	0.000623	0.000453	9.72e-06	2,175
8	0.000750	0.000477	1.01e-05	2,244
9	0.000755	0.000492	1.08e-05	2,078
10	0.000659	0.000441	9.83e-06	2,016
11	0.000720	0.000458	9.95e-06	2,122
12	0.000771	0.000504	1.20e-05	1,765
13	0.000678	0.000448	1.10e-05	1,654
14	0.000817	0.000519	1.28e-05	1,632
15	0.000745	0.000519	1.31e-05	1,579
16	0.000727	0.000533	1.38e-05	1,485
17	0.000742	0.000474	1.25e-05	1,447
18	0.000630	0.000426	1.21e-05	1,251
19	0.000515	0.000403	1.15e-05	1,238
20	0.000720	0.000509	1.35e-05	1,427
21	0.000577	0.000504	1.36e-05	1,378
22	0.000579	0.000434	1.25e-05	1,210
23	0.000775	0.000534	1.70e-05	989
24	0.000777	0.000501	1.42e-05	1,239
25	0.000670	0.000430	1.61e-05	713
26	0.000792	0.000579	1.85e-05	983
27	0.000860	0.000540	1.96e-05	757
28	0.000677	0.000477	1.50e-05	1,012
All	0.000712	—	—	48,438

Table S2.Per-individual genomic inbreeding coefficients for all 11 Arunachali Mithun individuals. F_{ROH} was derived from cumulative ROH length relative to autosomal genome length (detectRUNS); F_{HOM} from excess observed over expected homozygosity (PLINK --het); F_{UNI} (GCTA Fhat3) from the correlation between uniting gametes; F_{GRM} from the diagonal of the genomic relationship matrix (GCTA GRM). Individual IDs correspond to the sequential sample numbering used in the main manuscript. The mean row (blue) corresponds to the values reported in Table 4 of the main text.

Individual ID	F_{ROH}	F_{HOM}	F_{UNI}	F_{GRM}
1	0.0961	0.1957	0.1602	0.1068
2	0.1044	0.1771	0.1015	-0.0002
3	0.1276	0.1702	0.0819	-0.0386
4	0.1199	0.1207	0.1525	0.2061
5	0.1116	0.2259	0.2776	0.3618
6	0.1856	0.1403	0.0900	0.0502
7	0.1838	0.1605	0.2053	0.2551
8	0.1509	0.0433	0.1020	0.1825
9	0.2077	0.2891	0.3052	0.3318
10	0.1500	0.0535	0.0915	0.1392
11	0.1514	0.0440	0.0076	-0.0195
Mean	0.1445	0.1473	0.1432	0.1432

Note: Negative F_{GRM} values are mathematically expected for individuals whose genomic similarity to the population mean is below average; they do not indicate negative inbreeding in a biological sense.

Table S3.

Sensitivity analysis: comparison of key population genetic parameters between the full dataset (n = 11) and the unrelated subset (n = 9). Related individuals excluded from the n = 9 subset: 3 (PI_HAT = 0.715 with 2) and 11 (PI_HAT = 0.857 with 6). Remaining individuals: 1, 2, 4, 5, 6, 7, 8, 9, 10. Direction symbols: ↓ = decrease, ↑ = increase, ↔ = no meaningful change.

Parameter	n=11 (Full Dataset)	n=9 (Unrelated Subset)	Absolute Difference	% Change	Direction	Conclusion
1. Heterozygosity						
Observed Heterozygosity (H_o) - Mean	0.2854	0.2937	+0.0083	+2.8%	↑	Robust: negligible change
Expected Heterozygosity (H_e) - Mean	0.3347	0.3349	+0.0002	+0.06%	↑	Robust: virtually identical
H_o SD	0.1620	0.1669	+0.0049	+2.9%	↑	Robust: minor change
H_e SD	0.1207	0.1205	−0.0002	−0.2%	↓	Robust: no meaningful change
2. Nucleotide Diversity (π)						
Mean π (genome-wide)	7.16×10^{-4}	7.25×10^{-4}	$+0.09 \times 10^{-4}$	+1.3%	↑	Robust: within noise range
SD of π	4.87×10^{-4}	4.91×10^{-4}	$+0.04 \times 10^{-4}$	+0.8%	↔	Robust: negligible change
3. Inbreeding Coefficients (Mean ± SD)						
F_{ROH} — Mean	0.1445 ± 0.0364	0.1381 ± 0.0401	−0.0064	−4.4%	↓	Robust: slight reduction as expected
F_{HOM} — Mean	0.1473 ± 0.0783	0.1380 ± 0.0784	−0.0093	−6.3%	↓	Robust: modest reduction
F_{UNI} — Mean	0.1432 ± 0.0892	0.1361 ± 0.0654	−0.0071	−5.0%	↓	Robust: modest reduction
F_{GRM} — Mean	0.1432 ± 0.1384	0.1350 ± 0.0896	−0.0082	−5.7%	↓	Robust: consistent direction
4. LD Decay & Effective Population Size (N_e)						

LD half-decay distance (r^2 = 0.1 threshold)	~1.0 Mb	~1.0 Mb	0 Mb	~0%	↔	Robust: LD pattern preserved
Ne at ~5 generations ago	~160	~122	−38	−23.8%	↓	Robust: n=9 consistently lower (related individuals inflate LD-based Ne)
Ne at ~10 generations ago	~423	~304	−119	−28.1%	↓	Robust: directional trend preserved
Ne at ~50 generations ago	~1,761	~1,247	−514	−29.2%	↓	Robust: directional trend preserved
Ne at ~100 generations ago	~3,865	~3,629	−236	−6.1%	↓	Robust: directional trend preserved; all estimates order-of-magnitude only
5. Runs of Homozygosity (ROH)						
Mean total ROH length (Mb) per individual	348.5 Mb	326.0 Mb	−22.5 Mb	−6.5%	↓	Expected: related individuals had long shared ROH
Genome-wide ROH distribution pattern	Distributed across all 28 chr	Distributed across all 28 chr	—	—	↔	Robust: same chromosomal pattern
6. Principal Component Analysis						
PC1 variance explained	15.33%	15.33%	0%	0%	↔	Robust: consistent
PC2 variance explained	12.26%	12.26%	0%	0%	↔	Robust: consistent
Clustering pattern	No discrete clusters; continuous gradient	No discrete clusters; continuous gradient	—	—	↔	Robust: same interpretation
7. ADMIXTURE Analysis						

Best-supported K (lowest CV error)	K=1 (CV=1.040)	K=1 (CV=1.040)	—	—	↔	Robust: same conclusion: no substructure
